# Three-year survival and distribution of lymph node metastases in gastric cancer following neoadjuvant chemotherapy: results from a European randomized clinical trial

**DOI:** 10.1007/s00464-023-10278-5

**Published:** 2023-07-19

**Authors:** Nicole van der Wielen, Freek Daams, Riccardo Rosati, Paolo Parise, Jürgen Weitz, Christoph Reissfelder, Ismael Diez del Val, Carlos Loureiro, Purificación Parada-González, Elena Pintos-Martínez, Francisco Mateo Vallejo, Carlos Medina Achirica, Andrés Sánchez-Pernaute, Adriana Ruano Campos, Luigi Bonavina, Emanuele L. G. Asti, Alfredo Alonso Poza, Carlos Gilsanz, Magnus Nilsson, Mats Lindblad, Suzanne S. Gisbertz, Mark I. van Berge Henegouwen, Uberto Fumagalli Romario, Stefano De Pascale, Khurshid Akhtar, Miguel A. Cuesta, Donald L. van der Peet, Jennifer Straatman

**Affiliations:** 1 Department of Gastrointestinal Surgery, Amsterdam University Medical Center Location VU University Medical Center, De Boelelaan 1117, ZH 7F020, 1081 HV Amsterdam, The Netherlands; 2 Department of Clinical Epidemiology, Amsterdam University Medical Center, Amsterdam, The Netherlands; 3 Department of Gastrointestinal Surgery, San Raffaele Hospital, Milan, Italy; 4 Department of of Visceral-, Thoracic and Vascular Surgery, University Hospital Carl Gustav Carus, Dresden, Germany; 5 Department of Surgery, Universitätsmedizin Mannheim, Medical Faculty Mannheim, Heidelberg University, Heidelberg, Germany; 6 Department of Surgery, Hospital Universitario de Basurto, Bilbao, Spain; 7 Department of Surgery, Hospital Clínico Universitario de Santiago de Compostela, Santiago de Compostela, Spain; 8 Department of Surgery, Hospital de Jerez, Jerez de la Frontera, Spain; 9 Department of Surgery, Hospital Clínico San Carlos, Madrid, Spain; 10 Department of Surgery and Division of Foregut Surgery, IRCCS Policlinico San Donato, University of Milan, Milan, Italy; 11 Department of Surgery, Hospital del Sureste, Madrid, Spain; 12 Division of Surgery, Department of Clinical Science Intervention and Technology, Karolinska Institutet and Department of Upper Abdominal Diseases, Karolinska University Hospital, Stockholm, Sweden; 13 Department of Surgery, Amsterdam UMC, Location AMC, University of Amsterdam, Cancer Center Amsterdam, Amsterdam, The Netherlands; 14 Digestive Surgery, European Institute of Oncology - IRCCS - Milan, Milan, Italy; 15 Department of Surgery, Salford Royal NHS Foundation Trust, Manchester, UK; 16 Cancer Treatment and Quality of Life, Cancer Center Amsterdam, Amsterdam, The Netherlands

**Keywords:** Survival, Lymph node distribution, Minimally invasive gastrectomy

## Abstract

**Background:**

Adequate lymphadenectomy is an important step in gastrectomy for cancer, with a modified D2 lymphadenectomy being recommended for advanced gastric cancers. When assessing a novel technique for the treatment of gastric cancer, lymphadenectomy should be non-inferior. The aim of this study was to assess completeness of lymphadenectomy and distribution patterns between open total gastrectomy (OTG) and minimally invasive total gastrectomy (MITG) in the era of peri-operative chemotherapy.

**Methods:**

This is a retrospective analysis of the STOMACH trial, a randomized clinical trial in thirteen hospitals in Europe. Patients were randomized between OTG and MITG for advanced gastric cancer after neoadjuvant chemotherapy. Three-year survival, number of resected lymph nodes, completeness of lymphadenectomy, and distribution patterns were examined.

**Results:**

A total of 96 patients were included in this trial and randomized between OTG (49 patients) and MITG (47 patients). No difference in 3-year survival was observed, this was 57.1% in OTG group versus 46.8% in MITG group (*P* = 0.186). The mean number of examined lymph nodes per patient was 44.3 ± 16.7 in the OTG group and 40.7 ± 16.3 in the MITG group (*P* = 0.209). D2 lymphadenectomy of 71.4% in the OTG group and 74.5% in the MITG group was performed according to the surgeons; according to the pathologist compliance to D2 lymphadenectomy was 30% in the OTG group and 36% in the MITG group. Tier 2 lymph node metastases (stations 7–12) were observed in 19.6% in the OTG group versus 43.5% in the MITG group (*P* = 0.024).

**Conclusion:**

No difference in 3-year survival was observed between open and minimally invasive gastrectomy. No differences were observed for lymph node yield and type of lymphadenectomy. Adherence to D2 lymphadenectomy reported by the pathologist was markedly low.

Adequate resection with lymphadenectomy remains the core of curative treatment for patients with advanced gastric cancer in the era of peri-operative chemotherapy. In total gastrectomy, the Japanese Gastric Cancer Association (JGCA) guidelines recommend radical resection with adequate lymphadenectomy, consisting of a modified D2 lymph node dissection with a minimum of 15 lymph nodes. Taking into consideration that D2 lymphadenectomy leads to superior outcomes in survival in comparison to D1 resection [1]. D1 nodes include the tier one nodes: around the crus, in the lesser omentum, and around the gastro-epiploic artery and supra- and infrapyloric nodes. D2 includes the additional removal of tier two nodes: along the celiac trunk, left gastric artery, common hepatic artery, hepatic proper artery, and splenic artery [2]. In line with the Japanese Classification of Gastric Cancer (JCGC), the European Society for Medical Oncology (ESMO) recommends radical resection and adequate lymphadenectomy, if possible, in a multimodal treatment setting (i.e., peri-operative chemotherapy) [3, 4]. When assessing a novel surgical technique in gastric cancer, the quality of resection, which includes lymphadenectomy, should be non-inferior. Regarding gastrectomy the ESMO guidelines from 2022 state that laparotomy is an acceptable approach and a laparoscopic approach may be selectively proposed in expert hands [4]. Lymph node harvest remains an important factor for survival and prognostication, even in the era of neoadjuvant therapy [5].

The here presented study is a retrospective analysis on the data from the randomized clinical trial on open versus minimally invasive total gastrectomy (STOMACH trial) [6]. This randomized controlled trial comparing minimally invasive total gastrectomy with open total gastrectomy showed similar short-term results. Importantly, no differences were observed in overall lymph node yield and one-year survival, indicating that minimally invasive total gastrectomy (MITG) is non-inferior to open total gastrectomy (OTG) regarding short-term oncological quality of resection.

The aim of the present retrospective analysis is to investigate the 3-year survival and to assess the differences in harvested lymph node stations between the two operation modalities, along with the distribution of positive nodes in patients with peri-operative chemotherapy.

## Methods

The full protocol for the STOMACH trial, the peri-operative outcomes, quality of resection, and one-year survival were published previously. The medical ethical board of all participating hospitals approved the trial. Written informed consent was obtained from all participating patients at the beginning of the trial. The study protocol prescribed that the resection was performed en bloc and the surgeon marked all lymph node stations attached to the specimen. The pathologist was blinded for the type of surgery. Lymph node metastases were confirmed by histopathological results from the specimen [6, 7]. This trial was registered on April 28, 2014 at Clinicaltrials.gov with the identifier NCT02130726.

### Patients

**Fig. 1:**
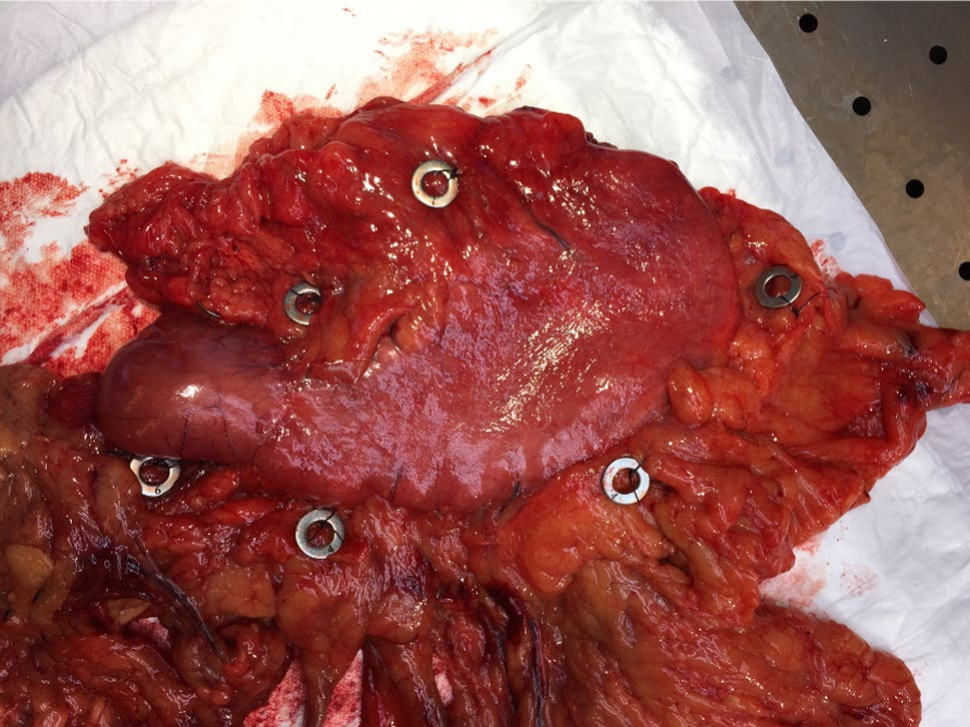
Resected specimen with attached markings

All patients included in this study had histologically proven clinically resectable gastric carcinoma (cT2-4a, N0-3, M0, or cT1N +) and underwent peri-operative chemotherapy. Patients were randomized to either OTG or MITG with a modified D2 lymphadenectomy. A picture of the resected stomach with the numbered tags corresponding the lymph node stations is depicted in Fig. 1. The details for preoperative workup, surgical procedures, and postoperative treatment were previously described [7].

### Outcomes

The primary outcome of this retrospective analysis was 3-year survival following OTG and MITG with modified D2 lymphadenectomy, in patients with gastric cancer, following chemotherapy. Furthermore, patterns in resected lymph node stations, the number of lymph nodes per lymph node station, and distribution pattern for lymph node metastases were assessed between OTG and MITG.

### Statistics

Statistical analysis was performed using SPSS statistical package, version 26 (IBM software). Lymph node dissection in OTG and MITG was compared as follows: Continuous variables were described as means and standard deviation for normal distributions and medians and interquartile ranges for non-normal distributions. Comparison tests were performed with Student’s *T* test and Mann–Whitney *U* tests as appropriate.

Frequencies were described as number and percentage of total. Comparison was performed with Chi-square tests; for variables with multiple categories additional testing within groups was performed with Bonferroni correction. A two-sided p-value of 0.05 was deemed statistically significant. Binary logistic regression techniques were used to assess predictors for an adequate D2 lymphadenectomy as determined by the pathologist.

## Results

### Patient characteristics

A total of 96 patients were included in this trial of which 49 patients were treated with OTG and 47 patients with MITG. Baseline characteristics are depicted in Table 1. No differences were observed in clinical TNM staging between OTG and MITG. Most patients had clinical T3 stage, 36 patients (73.4%) in the open group, and 30 patients (63.8%) in the minimally invasive group. Different neoadjuvant chemotherapy regimens were applied. Most patients received epirubicin, cisplatin, and capecitabine (ECC) or epirubicin, cisplatin, and fluorouracil (ECF) (42%), followed by fluorouracil, leucovorin, oxaliplatin, and docetaxel (FLOT) (19%). No differences in regimens were observed between OTG and MITG. Pathological outcomes are depicted in Table 2.

**Table 1:** Baseline characteristics

Baseline	OTG		MITG		*P*-value
	Mean	SD	Mean	SD	
Number of patients	49		47		
Age at time of surgery	61.8	10.0	59.4	12.5	0.298
Gender (male %)	32	65.3%	28	59.6%	0.674
ASA classification^a^					
ASA 1	6	12.2%	4	8.5%	0.813*
ASA 2	31	63.3%	30	63.8%	
ASA 3	12	24.5%	13	27.7%	
Tumor location					
Proximal	14	28.6%	12	28.9%	0.999*
Middle	25	51.0%	23	51.1%	
Distal	10	20.4%	9	20.0%	
Clinical T-stage					
T1	1	2.0%	2	4.3%	0.730*
T2	8	16.3%	9	19.1%	
T3	36	73.5%	30	63.8%	
T4	4	8.2%	6	12.8%	
Clinical N-stage					
N0	17	34.7%	17	36.2%	0.711*
N1	25	51.0%	26	55.3%	
N2	7	14.2%	4	8.5%	
Neoadjuvant chemotherapy					
ECC	12	24.5%	9	19.1%	0.690*
ECF	10	20.4%	9	19.1%	
EOX	13	26.5%	13	27.7%	
Folfox	0		2	4.3%	
FLOT	10	20.4%	8	17.0%	
Other	4	8.2%	6	12.8%	

*Additional testing within groups with Bonferroni correction showed no differences between groups

^a^American society of anesthesiologists (ASA)

**Table 2:** Pathology

Pathology	Open		Minimally invasive		*P*-value
Tumor type					
Intestinal-type adenocarcinoma	25	51.0%	16	34.0%	0.366*
Diffuse-type adenocarcinoma	19	38.8%	26	55.3%	
Carcinoid	1	2.0%	1	2.1%	
Signet cell carcinoma	3	6.1%	1	2.1%	
Other	1	2.0%	6	12.8%	
Pathological T-stage					0.937*
T0 (complete regression)	4	8.2%	3	6.4%	
Tis	2	4.1%	1	2.1%	
T1	8	16.3%	6	12.8%	
T2	4	8.2%	5	10.6%	
T3	19	38.8%	17	36.2%	
T4	12	24.5%	15	31.9%	
Pathological N-stage					0.323**
N0	23	46.9%	20	42.6%	
N1	13	26.5%	7	14.9%	
N2	8	16.3%	8	17.0%	
N3	5	10.2%	12	25.5%	
Number of examined LN per patient	44.3	± 16.7	40.7	± 16.3	0.209
N patients > 15 LN resected	48	98.0%	46	97.9%	0.742
Adherence to lymphadenectomy (based on pathology)					
< D1 lymphadenectomy	7	15%	3	6%	
D1 lymphadenectomy	14	30%	17	36%	
D1 + lymphadenectomy	12	26%	14	30%	
D2 lymphadenectomy	14	30%	13	28%	

There were no significant overall differences in pathological TNM staging, apart from more ypN3 patients being included in the minimally invasive group, namely 12 patients (25.5%) versus 5 patients (10.2%) in the open group. It should be noted that pathologists were blinded toward the surgical technique used in the patient.

### Survival

**Fig. 2:**
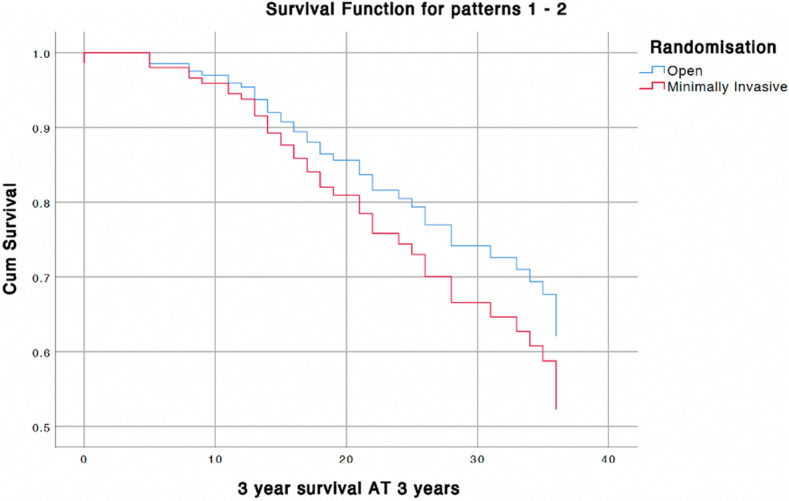
Survival curves corrected for ASA classification, age, pathological tumor, and node stage

No significant difference was seen in 3-year survival between both groups. Three-year survival was 57.1% in the open group versus 46.8% in the minimally invasive group, regardless of more N3 patients in the MITG group (*P* = 0.186). When corrected for ASA classification, age, pathological tumor, and node stage still no significant differences were observed (*P* = 0.357). An overview of corrected survival is depicted in Fig. 2.

### Lymphadenectomy

The mean number of examined lymph nodes per patients were 44.3 ± 16.7 in OTG and 40.7 ± 16.3 in MITG (*P* = 0.209). In 48 patients (98%) following OTG and in 46 patients (97.9%) following MITG more than 15 lymph nodes were resected (*P* = 0.742). Adequacy of lymph node dissection was compared in three ways: being type of dissection the surgeon reported (D0/D1/D2), the lymph node stations that the surgeon marked on the specimen, and whether the pathologist actually found lymph nodes in the marked station.

Although the intent was to perform a D2 lymphadenectomy in each patient, surgeons were asked to report which stations were actually resected following the procedure. When assessing the reported resected stations, according to the surgeon, adequate D2 resection was performed in 35 patients (71.4%) following OTG and in 35 patients (74.5%) following MITG. Analysis of type of lymphadenectomy per clinical disease stage revealed that a lower clinical stage was associated with not obtaining a D2 lymphadenectomy. In stage I disease, D2 lymphadenectomy was obtained in 36.4% of patients, versus above 70% for stages II and up (*P* = 0.009). Additional Cox-regression analysis showed no difference in survival between D0, D1, and D1 + versus D2 lymphadenectomy (*P* = 0.55). After resection, the surgeon marked each resected lymph node station before sending the specimen to the pathologist.

Upon pathological examination of these marked stations, lymph nodes were not always identified in the marked stations. When assessing the stations, which actually contained lymph nodes, it seems that only 14 patients (30%) following OTG and 17 patients (36%) following MITG had a complete D2 lymphadenectomy. Further statistical analysis revealed no difference in D2 lymphadenectomy rate per disease stage (*P* = 0.638).

Binary logistic regression for predictors of inadequate D2 lymphadenectomy showed that patient factors such as age, gender, BMI, and comorbidities were not associated with obtaining an inadequate pathological D2 lymphadenectomy. Regarding preoperative staging, a clinical N2 staging or higher was associated with a higher odds ratio of obtaining adequate lymphadenectomy (Odds ratio 3.54 with 95% confidence interval 1.079–12.82).

### Lymph nodes resected per lymph node station

All surgeons reported resection of the right paracardial, left paracardial, lesser curve, and infrapyloric lymph node stations. Almost all surgeons reported resection of the suprapyloric, left gastric artery, common hepatic artery, and celiac trunk lymph node stations. The lymph nodes at the splenic hilum was the least reported resected lymph node station, with 27 patients (57.4%) in the open group and 23 patients (48.9%) in the minimally invasive group. The difference between D1 + or D2 lymphadenectomy was mainly due to not resecting the lymph nodes in the splenic hilum. An overview of lymph node yield is depicted in Table 3.

**Table 3:** Lymph node yield

Lymph node (LN) yield	Open	MI	*P*-values
Tier one lymph nodes			
Lesser curvature			
Station 1–right cardia nodes			
Surgeon stated as resected (%)	100%	100%	NA
LN in station (%)	89.8%	95.7%	0.549
Average LN yield (*n*)	3 (2–6)	3 (2–6)	0.934
LN metastases in station (%)	11.6%	21.7%	0.262
Station 3–nodes along lesser curvature			
Surgeon stated as resected (%)	100%	100%	NA
LN in station (%)	91.8%	100.0%	0.387
Average LN yield (*n*)	5 (3–15)	3 (2–8)	0.019
LN metastases in station (%)	16.5%	18.7%	0.827
Station 5–suprapyloric nodes			
Surgeon stated as resected (%)	98.0%	95.7%	0.613
LN in station (%)	93.8%	97.8%	0.549
Average LN yield (*n*)	1 (0–3)	1 (0–3)	0.381
LN metastases in station (%)	2.3%	8%	0.089
Greater curvature			
Station 2–left cardiac nodes			
Surgeon stated as resected (%)	100%	100%	NA
LN in station (%)	89.8%	93.6%	0.610
Average LN yield (*n*)	2 (1–4)	2 (1–3)	0.181
LN metastases in station (%)	4.7%	17.8%	0.090
Station 4–nodes along the greater curvature			
Surgeon stated as resected (%)	100%	100%	NA
LN in station (%)	93.9%	100.0%	0.368
Average LN yield (*n*)	6 (2–11)	5 (2–12)	0.770
LN metastases in station (%)	13.2%	15.4%	0.817
Station 6–infrapyloric nodes			
Surgeon stated as resected (%)	100%	100%	NA
LN in station (%)	91.8%	97.9%	0.513
Average LN yield (*n*)	4 (2–9)	4 (2–7)	0.487
LN metastases in station (%)	11.4%	26.7%	0.104
Tier two lymph nodes			
Station 7–left gastric artery			
Surgeon stated as resected (%)	98.0%	97.9%	0.999
LN in station (%)	91.7%	95.7%	0.610
Average LN yield (*n*)	3.5 (2–7)	3.5 (1–6.5)	0.580
LN metastases in station (%)	9.3%	34.9%	0.008
Station 8–common hepatic artery			
Surgeon stated as resected (%)	98.0%	95.7%	0.613
LN in station (%)	95.8%	93.3%	0.147
Average LN yield (*n*)	2 (1–4)	2 (1–4.5)	0.842
LN metastases in station (%)	6.8%	16.7%	0.191
Station 9–celiac trunk			
Surgeon stated as resected (%)	100%	93.6%	0.113
LN in station (%)	79.6%	90.9%	0.589
Average LN yield (*n*)	2 (1–4)	4 (1–6)	0.125
LN metastases in station (%)	18.4%	27.5%	0.424
Splenic lymph nodes	Open	MI	p-values
Station 10–splenic hilum			
Surgeon stated as resected (%)	57.4%	48.9%	0.683
LN in station (%)	74.1%	91.3%	0.597
Average LN yield (*n*)	1.5 (0–4)	1 (0–4)	0.989
LN metastases in station (%)	5.0%	14.3%	0.606
Station 11–splenic artery			
Surgeon stated as resected (%)	89.4%	76.6%	0.302
LN in station (%)	83.3%	83.3%	0.327
Average LN yield (*n*)	2(0–3)	1 (0–3)	0.796
LN metastases in station (%)	5.9%	16.7%	0.238
Station 12–hepatoduodenal ligament			
Surgeon stated as resected (%)	83.0%	74.5%	0.631
LN in station (%)	71.8%	88.6%	0.520
Average LN yield (*n*)	1 (0–2)	2 (0–4)	0.096
LN metastases in station (%)	3.6%	13.8%	0.375

### Lymph node metastases per lymph node station

A division was made between tier one lymph nodes and tier two lymph nodes based on the anatomical location as described by the JGCA [1]. Tier one lymph nodes contain stations 1 to 6 and the tier two lymph nodes contain stations 7 to 12.

Tier one lymph node metastases were observed in 42.9% of patients in OTG and 53.3% of patients in MITG. This was not significantly different (*P* = 0.680). There was a significant difference in tier two lymph node metastases, namely 19.6% of patients in OTG versus 43.5% of patients in MITG (*P* = 0.024).

Further in-depth analysis per lymph node station revealed lymph nodes metastases from station 7 (left gastric artery) showed a significant difference between OTG and MITG, respectively, 9.3% versus 34.9% (*P* = 0.008). There was no difference in percentages in which patients this station was resected or the average lymph node yield between OTG and MITG. All other tier two lymph node stations (8–12) showed no significant difference between both groups. Assessment of distribution of lymph node metastases in proximal, middle, and distal tumors revealed no difference for each lymph node station, although it should be noted that the groups are rather small. An overview of lymph node metastases per lymph node station is depicted in Table 3.

**Fig. 3:**
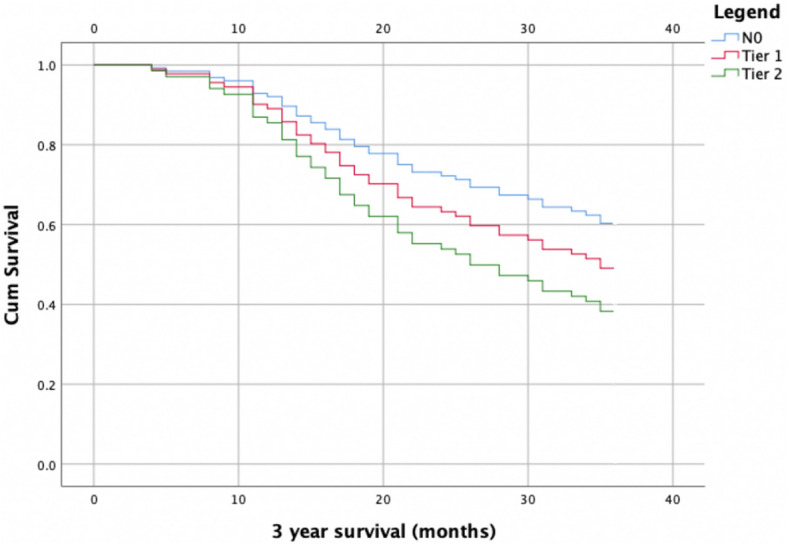
Cox-regression survival curves for patients with N0 disease, tier one lymph node metastases, and tier two nodal metastases. Correction was applied for T-stage and completeness of D2 resection

Assessment of survival via Cox-regression for tier one and tier two lymph nodes revealed no difference between N0 and tier one positive disease at 3 years (*P* = 0.303), Tier two positive lymph nodes were associated with decreased overall survival (*P* = 0.016). Correction was applied for completeness of D2 resection and cT-stage. Figure 3 depicts survival curves for patients with N0 disease, tier one, and tier two nodal metastases.

## Discussion

This retrospective analysis investigated three-year survival, lymphadenectomy, and potential differences in metastatic lymph node distribution between OTG and MITG for advanced gastric cancer after neoadjuvant chemotherapy. Results showed no difference in three-year survival between both groups. Additionally, no differences were observed between OTG and MITG for the number of resected lymph nodes and adequacy of lymphadenectomy performed, indicating that MITG is non-inferior to OTG regarding adequacy of oncological resection.

Interestingly, in 72.9% of cases, surgeons reported that a modified D2 lymphadenectomy was performed, whereas pathology results revealed that an adequate D2 lymphadenectomy, defined as lymph nodes present in all resected D2 stations, was only achieved in 32.3% of cases. Suggesting that even if stations are deemed resected according to anatomical landmarks, no lymph nodes may be present in the resected tissue. Alongside, the effect of neoadjuvant therapy on lymph node yield remains to be determined [8].

Several randomized trials reported non-compliance in D2 lymphadenectomy. In a large Dutch trial, conducted by the Dutch Gastric Cancer Group, randomization was set between D1 and D2 lymphadenectomy [9, 10]. Non-compliance and major non-compliance were seen in 80.5% and, respectively, 21.1% in the D1 group and in 81.6% and, respectively, 26% of the D2 lymphadenectomy group. In the randomized trial by the Italian Gastric Cancer group major non-compliance was 33.6% in the group allocated to a D2 dissection [11, 12]. Further emphasizing the need for a standardized approach in lymphadenectomy, preferably performed in specialized high-volume centers, for optimal results and adherence to D2 lymphadenectomy [4, 13].

A significant difference was seen in positive lymph nodes in station 7 (left gastric artery), 9.3% in the open group versus 34.9% in the minimally invasive group (*P* = 0.008). However, no differences were observed in the frequency of resection of station 7 nor the number of resected lymph nodes in this station between both groups. Therefore, it was concluded that this is based on a coincidence rather than a scientifical finding.

No differences in survival were seen between D2 lymphadenectomy and patients with D1 + lymphadenectomy or less, although it should be noted that the sample size was relatively small and type 2 errors cannot be excluded. However, overall survival at 3 years was worse for patients with tier two nodal metastases compared to patients with N0 or tier one nodal metastases, further underwriting the importance of an adequate D2 lymphadenectomy.

The results are in line with the trial by the Dutch Gastric Cancer Group, which reported no differences in 5-year survival between D1 and D2 lymphadenectomy. At 15-year follow-up disease-specific survival was significantly better for the D2 lymphadenectomy group, while overall survival remained similar [10, 11]. The effect of peri-operative chemotherapy on differences in survival between D1 + and D2 remains unclear.

Taking into account that patients in the MITG group more often had a N3 nodal status and more often had involvement of tier two nodes, no differences were observed in overall survival between OTG and MITG, even after correction for these factors.

A previous study, assessing prognostic factors based on clinicopathological outcomes, revealed a poorer prognosis in patients with N3 nodal involvement and patients with involvement of tier two lymph nodes. None of the patients in this study received peri-operative chemotherapy [14]. Long-term follow-up will give more insights into importance of lymphadenectomy in the era of peri-operative chemotherapy and the effect on survival in a European cohort.

One limitation of this study was that differences in type of lymphadenectomy was not a primary outcome in this trial and the patient groups might be too small to give an accurate conclusion. All resected lymph node stations were marked by the surgeon and further examined by the pathologist, giving important insights into difficulties and compliance with D2 lymphadenectomy in patients with gastric cancer treated with multimodality treatment. Several studies suggested that the overall survival is associated with the number of retrieved lymph nodes [15–17]. Whereas other studies focus more on the adequate resection of lymph nodes in certain anatomical locations [14]. Moreover, a larger lymph node yield further leads to more accurate staging [18].

Techniques for retrieval and marking of lymph nodes influence overall lymph node yield, for example, one study found that more lymph nodes were reported if a dedicated surgicopathological team assessed the specimen [19]. Further emphasizing the need for a standard operating procedure not only for lymphadenectomy but also for specimen handling and lymph node assessment. With the variation seen in lymph node yield, future studies should aim to assess adequate dissection by anatomical landmarks rather than number of lymph nodes found by pathologists.

## Conclusion

No difference in 3-year survival was observed between open or minimally invasive total gastrectomy. There were no differences regarding the number of dissected lymph nodes and type of lymphadenectomy between both groups. Assessment by the pathologist reported markedly low adherence to D2 lymphadenectomy. Overall survival was worse in patients who had tier two nodal involvement, even after correction for T-stage and completeness of D2 lymphadenectomy. Further underwriting the importance of a complete D2 lymphadenectomy.

Long-term survival data and analysis of lymphadenectomy reveal that minimally invasive gastrectomy is safe and non-inferior to open gastrectomy and may therefore be an alternative to an open technique.
