# Correction: Three-year survival and distribution of lymph node metastases in gastric cancer following neoadjuvant chemotherapy: results from a European randomized clinical trial

**DOI:** 10.1007/s00464-023-10336-y

**Published:** 2023-07-29

**Authors:** Nicole van der Wielen, Freek Daams, Riccardo Rosati, Paolo Parise, Jürgen Weitz, Christoph Reissfelder, Ismael Diez del Val, Carlos Loureiro, Purificación Parada-González, Elena Pintos-Martínez, Francisco Mateo Vallejo, Carlos Medina Achirica, Andrés Sánchez-Pernaute, Adriana Ruano Campos, Luigi Bonavina, Emanuele L. G. Asti, Alfredo Alonso Poza, Carlos Gilsanz, Magnus Nilsson, Mats Lindblad, Suzanne S. Gisbertz, Mark I. van Berge Henegouwen, Uberto Fumagalli Romario, Stefano De Pascale, Khurshid Akhtar, Miguel A. Cuesta, Donald L. van der Peet, Jennifer Straatman

**Affiliations:** 1 Department of Gastrointestinal Surgery, Amsterdam University Medical Center Location VU University Medical Center, De Boelelaan 1117, ZH 7F020, 1081 HV Amsterdam, The Netherlands; 2 Department of Clinical Epidemiology, Amsterdam University Medical Center, Amsterdam, The Netherlands; 3 Department of Gastrointestinal Surgery, San Raffaele Hospital, Milan, Italy; 4 Department of of Visceral-, Thoracic and Vascular Surgery, University Hospital Carl Gustav Carus, Dresden, Germany; 5 Department of Surgery, Universitätsmedizin Mannheim, Medical Faculty Mannheim, Heidelberg University, Heidelberg, Germany; 6 Department of Surgery, Hospital Universitario de Basurto, Bilbao, Spain; 7 Department of Surgery, Hospital Clínico Universitario de Santiago de Compostela, Santiago de Compostela, Spain; 8 Department of Surgery, Hospital de Jerez, Jerez de la Frontera, Spain; 9 Department of Surgery, Hospital Clínico San Carlos, Madrid, Spain; 10 Department of Surgery and Division of Foregut Surgery, IRCCS Policlinico San Donato, University of Milan, Milan, Italy; 11 Department of Surgery, Hospital del Sureste, Madrid, Spain; 12 Division of Surgery, Department of Clinical Science Intervention and Technology, Karolinska Institutet and Department of Upper Abdominal Diseases, Karolinska University Hospital, Stockholm, Sweden; 13 Department of Surgery, Amsterdam UMC, Location AMC, University of Amsterdam, Cancer Center Amsterdam, Amsterdam, The Netherlands; 14 Digestive Surgery, European Institute of Oncology - IRCCS - Milan, Milan, Italy; 15 Department of Surgery, Salford Royal NHS Foundation Trust, Manchester, UK; 16 Cancer Treatment and Quality of Life, Cancer Center Amsterdam, Amsterdam, The Netherlands

**Correction: Surgical Endoscopy** **https://doi.org/10.1007/s00464-023-10278-5**

The original online version of this article was revised to correct the information for affiliation 10. The correct information is: Department of Surgery and Division of Foregut Surgery, IRCCS Policlinico San Donato, University of Milan, Milan, Italy. The article was also updated to reflect that author Luigi Bonavina has this affiliation.

The original article has been corrected.

